# Indents

**Published:** 1901-11-15

**Authors:** 


### INDENTS.

☞ **Brief Articles of Technical or Scientific Value will receive notice and comment. Contributions invited.**

SOFT SOLDERING POWDER.—Take of granulated soft solder three parts, powdered sal ammoniac one part, and pulverized resin one part. Mix.—*Druggists' Circular.*

TO SET A LOGAN CROWN.—The ideal way, in my mind, of adjusting the Logan crown, is the Stowell method. I do not attempt to adjust the root of the crown, but I make an absolute joint everywhere. I make a cap over the stump of the root, either with or without a band, and set a pin in the cap. If it is a single-rooted, make one pin, and if it is the first superior bicuspid, put in two. I do not pay any attention to the Logan crown in making that cap. Then take the crown and cut the pin off and grind exactly to fit the cap and the articulation of the opposing teeth. Then take the crown and fill in the concavity with pure gold, and pressing it down, it spreads all over the bed of the crown. Then grind it again and put it in the mouth, put Parr's flux between, spat it down, take it out, invest and solder. That is the ideal way of adjusting a Logan crown. In order to get the proper alignment you must usually cut the pin off, or else you would have to cut the root to the crown. There need be no fear but that the solder will go to all portions and make it even stronger than the pin alone. With proper precautions taken, proper investing, proper flux used, and the proper heat, I

have never seen one of them break.—Dr. Patterson, in *Western Journal.*

The Value of a Right Attitude Toward Patients.—In all operations which we have, as dentists, to perform for our patients, there are none of them but what are better done and more comfortably to the patient with the use of a careful, considerate line of suggestion before beginning. This is true even where anesthesia is used. Patients who are properly prepared for it take an anesthetic much more easily and comfortably than those who are placed upon the operating-table without any preparation whatever in a mental way. We should bear constantly in mind that we are never free from suggestion and its influence. It has been said that we are a part of all that we have met. This is but an expression of a principle; and if our relation with and attitude toward our patient is constantly that of a gentle, easy, composed, well-in-hand character, it reflects upon them, and they reap the benefit of what we have attained ourselves. We have long been looking for a perfect local anesthetic and obtundent; they have not yet been found. The ones we have can be made a great deal more effective by suggestion. The patient is in the chair. You are ready to begin an operation. Be gentle in the application of the rubber dam. Use in most cases silk ligatures; they are more comfortable than clamps.—Dr. F. O. Hetrick, in *Western Journal.*

